# Adapting Training and Use of an Application for Cognitively Impaired Older Adults Amidst COVID-19

**DOI:** 10.1093/geroni/igab046.2479

**Published:** 2021-12-17

**Authors:** Francesca Falzarano, Sarah Weingast, Gabriel Gladstone-Groth, Marco Ceruso, Sara Czaja

**Affiliations:** 1 Weill Cornell Medicine, Douglaston, New York, United States; 2 Weill Cornell Medicine, Weill Cornell Medicine, New York, United States; 3 Weill Cornell Medicine, New York, New York, United States; 4 Weill Cornell Med, New York, New York, United States; 5 Weill Cornell Medicine/Center on Aging and Behavioral Research, New York, New York, United States

## Abstract

The emergence of COVID-19 and social distancing requirements have resulted in disruptions to daily life, reduced opportunities for social engagement, and diminished resource access for millions of older adults. Individuals with cognitive impairments (CI) are particularly vulnerable to risk for social isolation. This presentation will discuss the PRISM-CI pilot trial, which aims to examine the feasibility and potential efficacy of the PRISM-CI software system on enhancing connectivity and quality of life among a diverse sample of 50 older adults aged 65 and over with a CI. PRISM-CI, adapted from the PRISM system (developed by the Center for Research and Education on Aging and Technology Enhancement) for this population, is intended to support social engagement, memory, and access to resources and information. We will present data regarding the feasibility and perceived value of PRISM-CI and discuss the challenges, and strategies used, to adapt the PRISM-CI trial during the pandemic. We used a multi-modal approach to provide remote training and specialized tablet instruction that includes individualized training sessions tailored to individuals’ learning needs, hobbies, and prior technology use. The adapted protocol also involves the use of remote access software for troubleshooting. We will also discuss how participant feedback guided the inclusion of additional features, such as Zoom videoconferencing and virtual library access, for the PRISM-CI application. Finally, we will demonstrate how the adaptation of the PRISM-CI protocol holds promise for the use of flexible, remote technology approaches to reach socially isolated older adults to foster psychosocial well-being.

